# Oxidative stress at low levels can induce clustered DNA lesions leading to NHEJ mediated mutations

**DOI:** 10.18632/oncotarget.8298

**Published:** 2016-03-23

**Authors:** Vyom Sharma, Leonard B. Collins, Ting-huei Chen, Natalie Herr, Shunichi Takeda, Wei Sun, James A. Swenberg, Jun Nakamura

**Affiliations:** ^1^ Department of Environmental Sciences and Engineering, Gillings School of Global Public Health, Chapel Hill, NC 27599, USA; ^2^ Department of Genetics, The University of North Carolina at Chapel Hill, Chapel Hill, NC 27599, USA; ^3^ Department of Radiation Genetics, Graduate School of Medicine, Kyoto 606–8501, Japan

**Keywords:** oxidative stress, NHEJ, mutations, clustered DNA lesions, double strand breaks

## Abstract

DNA damage and mutations induced by oxidative stress are associated with various different human pathologies including cancer. The facts that most human tumors are characterized by large genome rearrangements and glutathione depletion in mice results in deletions in DNA suggest that reactive oxygen species (ROS) may cause gene and chromosome mutations through DNA double strand breaks (DSBs). However, the generation of DSBs at low levels of ROS is still controversial. In the present study, we show that H_2_O_2_ at biologically-relevant levels causes a marked increase in oxidative clustered DNA lesions (OCDLs) with a significant elevation of replication-independent DSBs. Although it is frequently reported that OCDLs are fingerprint of high-energy IR, our results indicate for the first time that H_2_O_2_, even at low levels, can also cause OCDLs leading to DSBs specifically in G1 cells. Furthermore, a reverse genetic approach revealed a significant contribution of the non-homologous end joining (NHEJ) pathway in H_2_O_2_-induced DNA repair & mutagenesis. This genomic instability induced by low levels of ROS may be involved in spontaneous mutagenesis and the etiology of a wide variety of human diseases like chronic inflammation-related disorders, carcinogenesis, neuro-degeneration and aging.

## INTRODUCTION

Most cancers result from an accumulation of genetic and chromosomal mutations caused by various endogenous or exogenous factors. Oxidative stress is one such major factor, with mutagenesis through oxidative DNA damage having been associated with spontaneous mutations, cancer, and aging [1–3]. The hydroxyl radical, the most biologically active free radical, is the predominant reactive oxygen species (ROS) that targets DNA. Although the hydroxyl radical resulting from the Fenton reaction of hydrogen peroxide (H_2_O_2_) and iron (II) has been linked with cancer [1, 4, 5], the exact mechanism by which oxidative stress induces mutagenesis is still unclear. For example, it has been well accepted that oxidative stress frequently induces 8-oxodG, which efficiently causes G to T base substitution mutations as determined by oligonucleotide-based bypass analysis and plasmid-based site-directed mutagenesis analysis [6, 7]. However, most human tumors are characterized by large genome rearrangements [8]. In addition, oxidative stress induced by glutathione depletion has been reported to cause DNA deletion in mice [9]. Glutathione is a major antioxidant which detoxifies H_2_O_2_ and helps maintain the reduced state of many proteins necessary for their function. In the absence of glutathione, the levels of ROS would rise creating an imbalance and subsequently oxidative stress. These previous results suggest that ROS may cause gene and chromosome mutations through DNA double strand breaks (DSBs), one of the most severe forms of DNA damage. DSBs, if left unrepaired or misrepaired, can cause cell death, chromosome instability, and cancer. However, the generation of DSBs by low levels of ROS is still controversial partly due to technical problems (*e.g.* the neutral Comet assay [10] and/or the usage of non-specific biomarker for DSBs such as γ-H2AX) [11, 12]. Neutral Comet assay appears to be more sensitive than the PFGE assay although the specificity of the assay is a concern due to the migration of unwound DNA resulting from SSBs [10]. This could be particularly true in the presence of H_2_O_2_ because of the efficient formation of SSBs over DSBs (2000 to 1 ratio) [13]. γ-H2AX foci have been reported as a reliable and accurate marker for DNA DSBs following ionizing radiation [14]; however, in the case of H_2_O_2_ treatment, γ-H2AX foci were reported to be distinct from those caused by ionizing radiation and were found to be heterogeneous in nuclei without a direct correlation with the amount of DSBs [15]. Therefore, the phenomenon of DSBs formation as a result of low levels of oxidative stress in cells and its role in mutagenesis have yet to be elucidated.

To counter DSBs and other types of oxidative DNA damage, mammalian cells possess DNA repair systems that serve to prevent and repair ROS-induced damage. Some of these DNA repair pathways in cells do not possess high fidelity, leading to genomic instability. Interestingly, error-prone non-homologous end joining (NHEJ) repair-deficient cells were found to be hyper-sensitive to high cellular oxygen tension, suggesting that even endogenous ROS can cause DSBs at the cellular level [16]. However, little is known about the comparative contribution of each repair protein/pathway in preventing mutations caused by oxidative stress. DSBs can also arise as repair intermediates during the processing of oxidatively induced clustered DNA lesions (OCDLs) [17]. OCDLs are closely spaced lesions (within 20 bp) in the form of abasic (AP) sites, oxypyrimidines, oxypurines and single strand breaks [18]. Previously, Chastain et al. [19] have reported presence of closely spaced abasic sites (e.g., 20 AP sites in a 6 kb length of DNA). These sites were detected using DNA fiber assay. These AP site clusters are different than OCDLs in terms of distance between lesions and based on the fact that OCDLs can have a combination of different DNA lesions including abasic sites, SSBs and oxidative damaged bases. OCDLs can be detected using pulse field gel electrophoresis (PFGE) while in DNA fiber assay the resolution (within 20 bp) might be an issue. These OCDLs, compared to single DNA lesions, can be challenging for the cells to repair, making them more toxic and mutagenic [20]. Although it is well reported that high energy ionizing radiation is a strong inducer of OCDLs, the formation of OCDLs as a result of low levels of oxidative stress in cells and their subsequent role in mutagenesis are still unknown. Therefore, in the present study, we have addressed whether physiologically-relevant levels of ROS cause OCDLs and DSBs. We also investigated the comparative contribution of each repair protein/pathway in the DNA damage response and in mutagenesis caused by oxidative stress.

## RESULTS AND DISCUSSION

### Non-homologous end joining (NHEJ) and homologous recombination (HR) confer tolerance to H_2_O_2_-induced oxidative DNA damage

**Figure 1:**
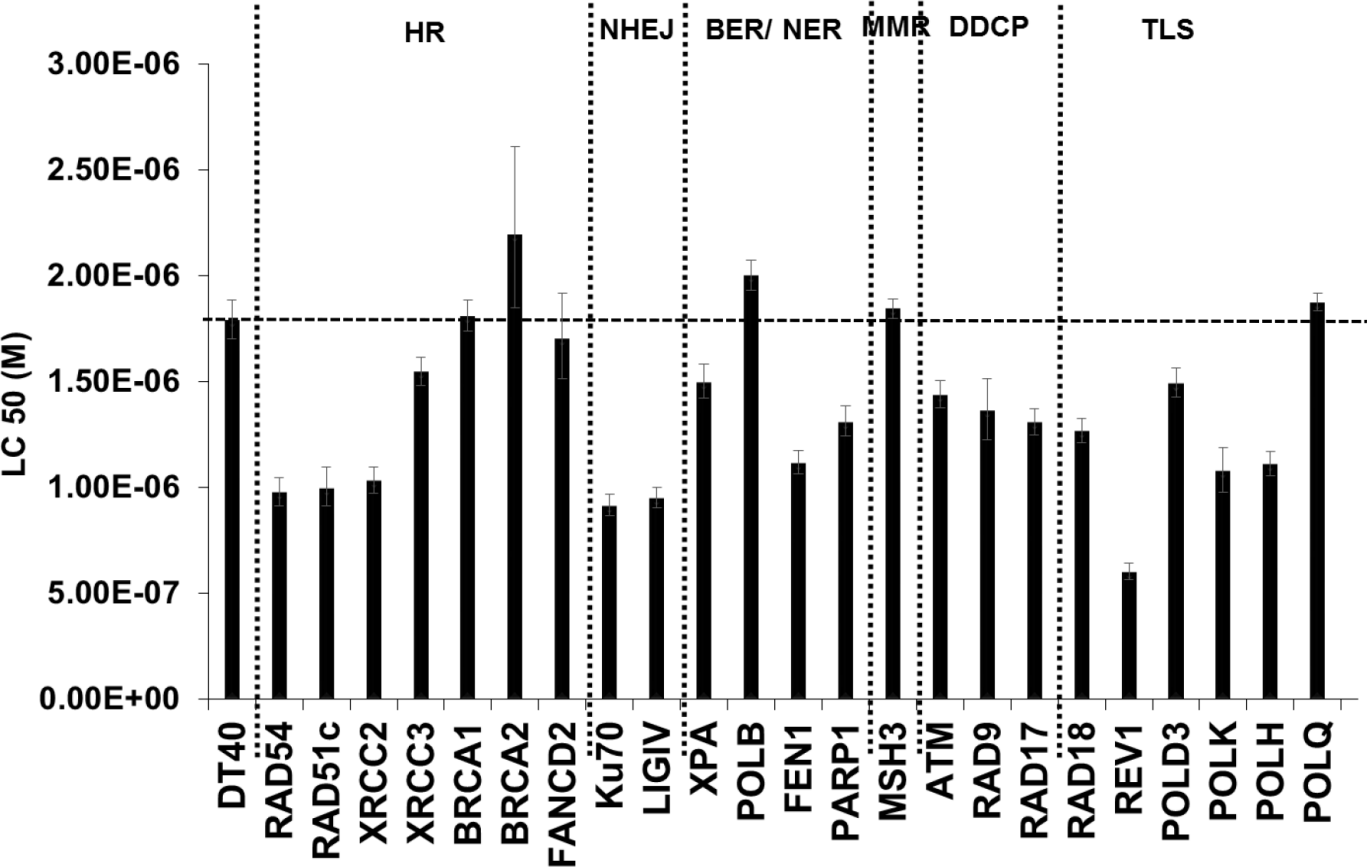
DNA damage response analysis in DT40 cells and their DNA repair mutants exposed to H_2_O_2_ Cells were exposed to different concentrations of freshly prepared H_2_O_2_ at 37°C. Survival data were log-transformed giving approximate normality. Analysis of covariance (ANCOVA) was used to test for mean intercept differences and differences in the slopes of the linear dose response curves between wild type and a series of mutant cells. Error *bars* indicate the 95% *confidence intervals. HR- homologous recombination; NHEJ- Non-homologous end joining; BER/ NER- Base excision repair/ Nucleotide excision repair; MMR- Mismatch repair; DDCP- DNA damage checkpoint; TLS- Translesion synthesis*.

We first performed an extensive DNA damage response analysis using a reverse genetic approach in order to identify the critical DNA repair pathways activated upon low doses of oxidative stress. The sensitivity of wild type DT40 cells and an array of their isogenic DNA repair mutant cells (Supplementary Table S1) to low concentrations H_2_O_2_ was compared (Figure 1) and mutant cell lines showing a statistically significant reduction in LC50 compared to the parental cell line were considered hypersensitive. Hypersensitivity of *RAD54, RAD51c, XRCC2, KU70, LIGIV, FEN1, REV1, POL kappa*, and *POL eta*-deficient cells to H_2_O_2_ indicated that base excision repair (BER), translesion synthesis (TLS), homologous recombination (HR) and NHEJ are essential for tolerating oxidative DNA damage. An interesting observation was the hypersensitivity of not only HR-deficient cells but also NHEJ knock-out cells to H_2_O_2_, in addition to toxicity in ATM-deficient cells, suggesting that DSBs may play a key role in low dose H_2_O_2_ toxicity. Hypersensitivity of cells deficient in *KU70, LIGIV* implies that H_2_O_2_ can induce DSBs leading to error-prone NHEJ repair processes. Similar hypersensitivity to H_2_O_2_ has been reported in *Ku80*-deficient mouse embryonic fibroblasts exposed to H_2_O_2_, indicating that the NHEJ pathway is also critical in tolerating oxidative DNA damage in mammalian cells [21]. However, it is not clear if H_2_O_2_ can induce any direct or indirect DSBs, especially at low levels. This directed us to investigate the formation and repair of DSBs in cells exposed to low concentrations of H_2_O_2_. In the present study, we have chosen very low concentrations of H_2_O_2_ that are physiologically and biologically relevant especially during inflammatory processes [22–24]. These low exposure concentrations are biologically more relevant compared to very high concentrations of H_2_O_2_ used in some previous studies [25, 26] that do not occur in the cellular and molecular environment.

**Figure 2:**
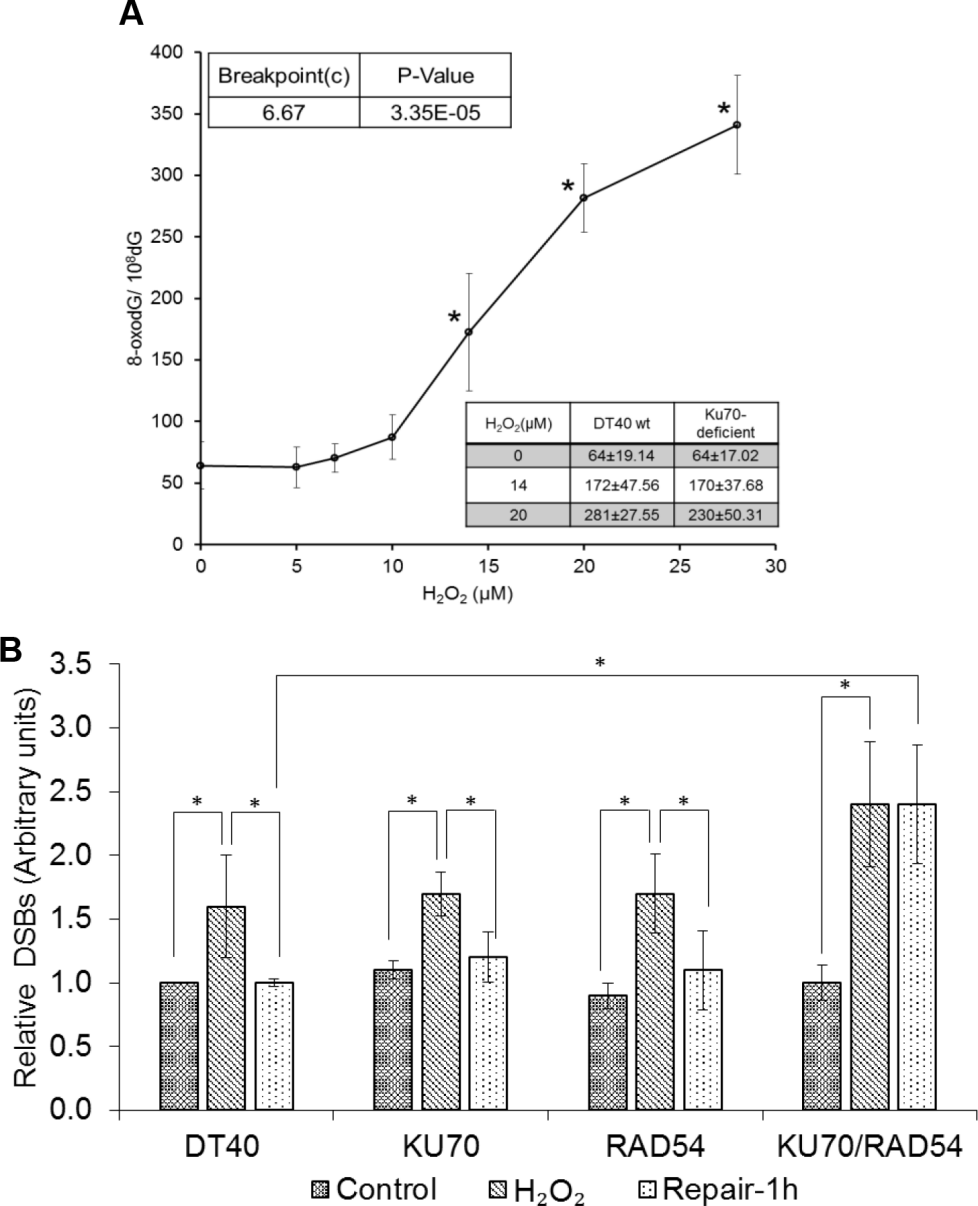
DNA damage induced by low levels of H_2_O_2_ (**A**) 8-oxodG amounts in DT40 cells exposed to H_2_O_2_ for 30 min. The box shows 8-oxodG amounts in isogenic *KU70*-deficient cells under similar conditions. Data represent mean ± SD. One-way analysis of variance (ANOVA) with Dunnet's test was employed to test for statistically significant differences compared to control. In a second type of statistical analysis, a piece-wise linear model (the hockey stick model) was applied to find the breakpoint value. (**B**) Generation and repair of DSBs in DT40 and isogenic DSBs repair-deficient cells. To understand the repair kinetics, the H_2_O_2_ dose was increased to 50 μM for repair experiments. Cells were exposed to H_2_O_2_ for 30 min and processed further for PFGE. Bands representing DSBs were quantified using Quantiscan (Biosoft, Cambridge, UK). Relative DSB levels were obtained by comparing DSBs signals in each sample to the background signals observed for unexposed wild type DT40 cells. Data represent mean (*n* = 5) ± SD. *p < 0.05 when compared using Student's *t* test.

8-oxodG (8-hydroxy-2′-deoxyguanosine) is one of the most frequently utilized biomarkers of oxidative DNA damage due to its strong correlation with ROS production. We measured 8-oxodG levels in cells exposed to H_2_O_2_ using LC-MS/MS to understand the occurrence of oxidative DNA damage at these low doses. It is important to note that H_2_O_2_ concentrations used for 8-oxodG analysis are ~10 times higher than LC50 levels in our DDR analysis (Figure 2A). This is due to the difference of cell concentrations (1 million cells/mL *vs* 0.01 million cells/mL) and exposure duration (30 min *vs* continuous) between two different assays (8-oxodG *vs* DDR). The extent of oxidative stress depends on the cell concentrations during H_2_O_2_ exposure [27]. Since large numbers of cells are needed to detect DNA damage, we used higher concentrations of DT40 cells and H_2_O_2_ for 8-oxodG analysis as compared to DDR analysis. Indeed, we detected a non-linear increase in 8-oxodG levels with a breaking point (departure points) from endogenous levels of 8-oxodG at 6.7 μM (Figure 2A). This indicates that low levels of H_2_O_2_ are enough to induce oxidative DNA damage in DT40 cells.

### H_2_O_2_ induces DNA DSBs in non-replicating cells through oxidative clustered DNA lesions (OCDLs)

The DSBs formed by exposure to H_2_O_2_ were assessed using pulse field gel electrophoresis (PFGE). To rule out the possibility of any artifact generated by depurination at high temperatures or oxidative stress during PFGE sample preparation, we performed lysis at a low temperature (4°C) and used a radical trapping agent (TEMPO) to quench free radicals. To understand the repair kinetics, the H_2_O_2_ dose was increased to 50 μM for repair experiments (Figure 2B). Wild type DT40 cells or cells with at least one major DSB repair pathway (*KU70* or *RAD54* deficiency) showed the repair of DSBs after 1 h exposure. However, combined deficiency of both major pathways (*KU70*/*RAD54* double knock-out) resulted in almost unaltered DSBs levels after 1 h following H_2_O_2_ exposure (Figure 2B). This shows that DSB repair is essential to counter H_2_O_2_ induced oxidative lesions. This data also suggests, that NHEJ can act as backup pathway in the absence of HR and vice-versa. However, in the absence of both, the repair of H_2_O_2_-induced DSBs becomes difficult. DT40 cells and their isogenic *KU70*, *RAD54* and *KU70/RAD54* double knock-out cells were also exposed to lower H_2_O_2_ concentration (20 μM) and showed a significant increase in DSBs (data not shown). One limitation of PFGE method is the low sensitivity for detecting persistent DNA DSBs [12]. For example, for X-rays at least 2–5 Gy of exposure is required to see some signal unlike radiation induced foci (53BP1 or γH2AX) which can go down to much lower sensitivities [28, 29]. This lower sensitivity might be the reason for the low signal of DSBs observed here at low doses of H_2_O_2_ when assessed by PFGE.

**Figure 3:**
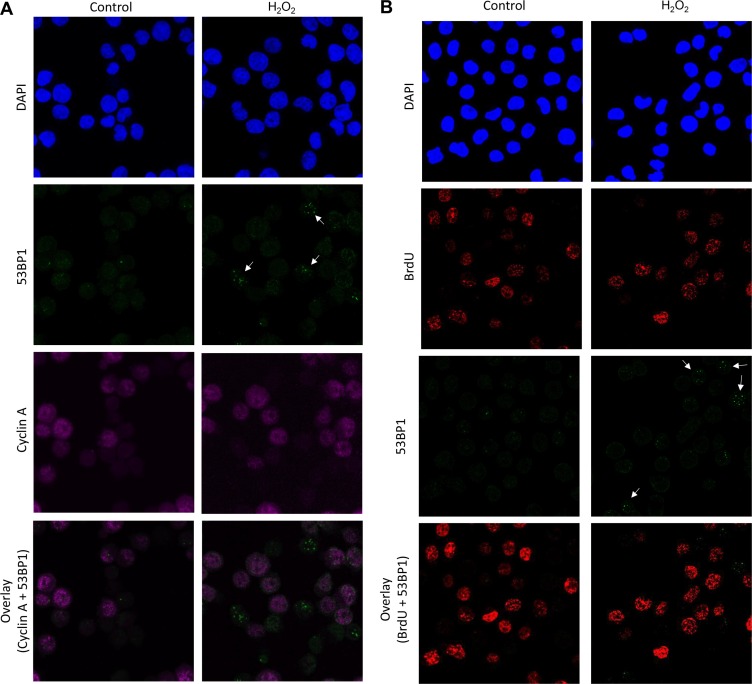
Induction of 53BP1 foci in G1 cells following exposure to H_2_O_2_ (**A**) TK6 cells were exposed to H_2_O_2_ (40 μM) for 30 min and co-immunostained with anti-53BP1 (green) and anti-cyclin A (magenta). (**B**) TK6 cells were pulse-labelled with BrdU for 15 min and then further incubated with H_2_O_2_ (40 μM) for 30 min. Cells were fixed and labelled with anti-53BP1 (green) antibody. BrdU-labelled cells were visualized with Alexa Fluor 555 (red) antibody. Nuclei were counterstained with DAPI (blue). The images were recorded using confocal microscopy and represents maximum intensity projections of z-stacks. 53BP1 foci are marked by white arrows. Magnification, 600×.

Apart from PFGE, we employed another technique to further confirm the induction of DSBs. We examined nuclear 53BP1 foci formation, a marker for DSBs, in TK6 cells after 30 min exposure to H_2_O_2_. A clear and increased 53BP1 foci formation was observed in H_2_O_2_ exposed cells compared to control (Figure 3A). To find out if these DSBs are a result of SSBs/ oxidative DNA damage encountering replication fork, we performed co-immunostaining of TK6 cells with 53BP1 and cyclin A. Cyclin A is mainly present in the S and G2 phase with slowly disappearing during the course of mitosis. Interestingly, majority of the 53BP1 foci were present in the G1-phase cells as indicated by the cyclin A negative staining. This was further confirmed by the BrdU pulse-labelling of cells just before the H_2_O_2_ treatment to label S-phase cells. Again most of the 53BP1 were found to present in the BrdU-negative cells indicating that the DSBs are arising independent of replication (Figure 3B). The possibility that the foci observed in the present study were generated in the S-phase cells and then transmitted and detected in the subsequent cell cycle phases including G1 can be ruled out due to very short exposure/ experiment time (30 min) used in our study. A previous study by Yang et al. has also demonstrated similar observations of H_2_O_2_ -mediated DSBs occurring independent of replication, although the concentrations of H_2_O_2_ used were much higher and not biologically relevant [25]. In addition, the neutral Comet assay was used to assess the DSBs which is not specific to DSBs and can detect SSBs as well [10]. The present data also excludes the possibility that topoisomerase I mediated DNA damage during replication is the main cause of DSBs observed in this study. TDP1 (tyrosyl-DNA phosphodiesterase 1) is an enzyme that is involved in repairing stalled topoisomerase I-DNA complexes by catalyzing the hydrolysis of the phosphodiester bond between the tyrosine residue of topoisomerase I and the 3-prime phosphate of DNA. This protein may also remove glycolate from single-stranded DNA containing 3-prime phosphoglycolate, suggesting a role in repair of free-radical mediated DNA double-strand breaks. In fact, we also compared the DSBs level by PFGE between the TDP1 deficient cells compared to the parental wild type cells, however no increase in the DSBs were observed in the absence of TDP1 (data not shown). We could not perform immunofluorescence experiments in the DT40 cells due to non-availability of antibodies specific to these cells.

This must be mentioned here that the γH2AX can be non-specific and unlike radiation the H_2_O_2_ does not induce the formation of distinct γH2AX foci but rather a whole nucleus staining pattern (pan-nuclear staining) with only few separate countable foci [15]. However, despite this we tried evaluating the co-localization of γH2AX with 53BP1. Although there was a presence of a nuclear wide γH2AX staining, we could see 53BP1 foci superimposing on some fractions of discrete γH2AX foci (data not shown) confirming the presence of true double strand breaks. Together, these observations indicate H_2_O_2_ at low levels causes’ double strand breaks formation predominantly in the G1-phase.

Pospelova et al. [30] showed that DDR response may be activated without any significant evidence of DNA damage e.g. in case of cellular senescence. In this pseudo-DDR response, while there was typical formation of γH2AX, while 53BP1 foci were absent. In addition the ATM foci showed a non-focal, diffuse pattern and there were no detectable strand breaks in the Comet assay. This possibility of pseudo-DDR can be ruled out in the present study, as there was a clear formation of 53BP1. Moreover, we also used PFGE assay, which is a direct approach to analyze DSBs formation.

**Figure 4:**
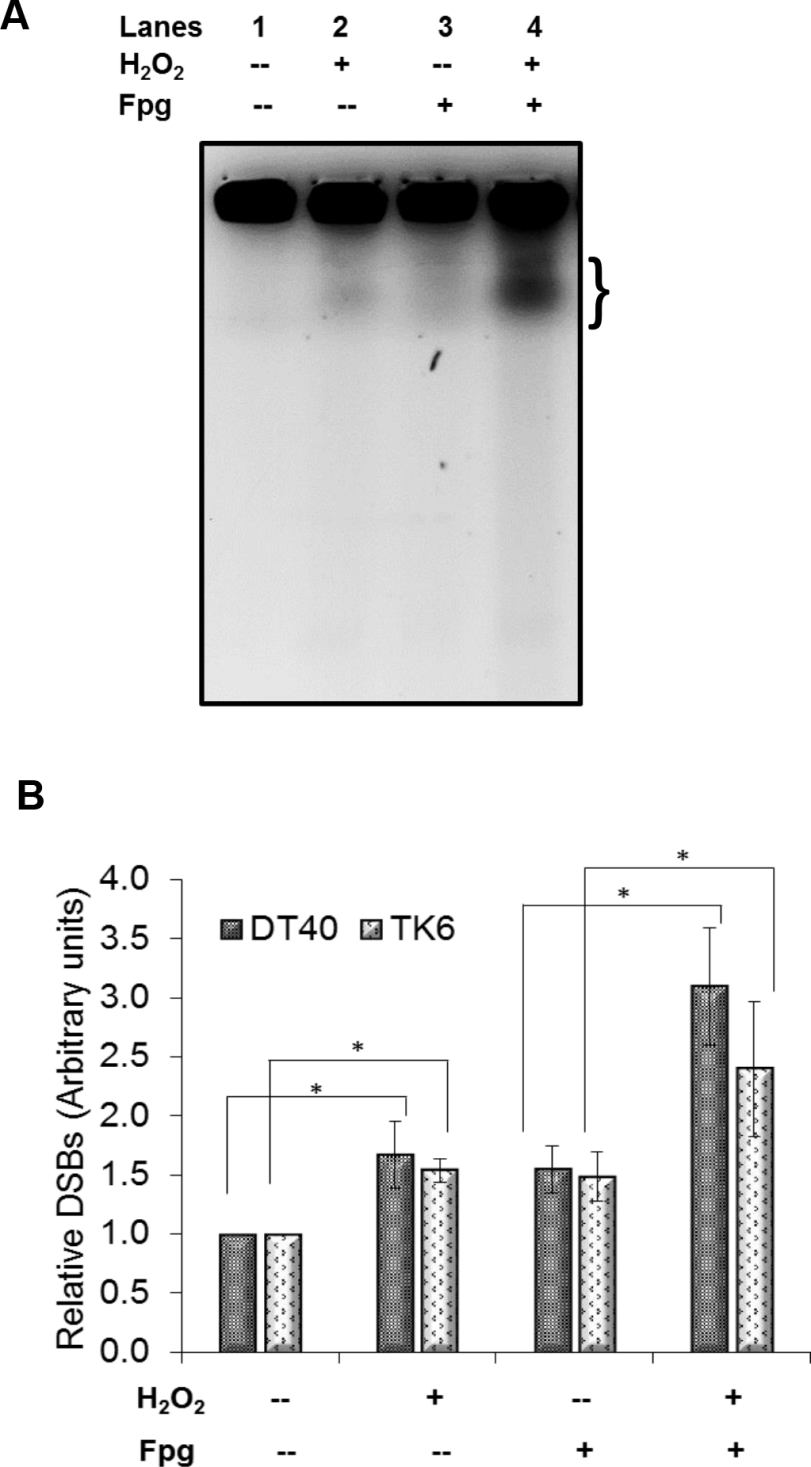
Formation of oxidatively induced clustered DNA lesions (OCDLs) on exposure to low doses of H_2_O_2_ (**A**) A representative image of OCDLs induced by 20 μM H_2_O_2_ in DT40 cells as assessed by modified PFGE. The area quantitated under each band (representing total DSBs) is shown by bracket (}). (**B**) Quantitative analysis of OCDL induction in DT40 and TK6 cells. Bands representing total DSBs were quantified using Quantiscan (Biosoft, Cambridge, UK). Relative DSB levels were obtained by comparing DSBs signals in each sample to the background signals observed for unexposed wild type DT40 cells. *p < 0.05; a statistical comparison of DSBs/OCDLs induction between exposed and unexposed cells under similar conditions (with/without enzyme) was conducted using a Student's *t*-test. Data represent mean (*n* = 3) ± SD.

Our findings above are interesting as they are in contrast to the generally accepted theory of H_2_O_2_ induced S-phase DSBs caused by SSBs/oxidative DNA damage encountering a replication fork. We also observed that in the absence of NHEJ which is the main pathway for DSB repair during G1, the cells are more sensitive to H_2_O_2_ than the parental DT40 cells (Figure 1). Therefore based on these two observations, we hypothesized that H_2_O_2_ at these concentrations may cause oxidatively induced clustered DNA lesions (OCDLs) in cells, which in turn can lead to DSBs with complex DNA ends. Bistranded OCDLs are closely spaced lesions in the form of abasic sites, oxypyrimidines, oxypurines and single strand breaks [18]. These OCDLs can be converted to DSBs during the repair process when both strands are incised simultaneously in close proximity. To determine the presence of OCDLs in H_2_O_2_-treated cells, PFGE was performed in combination with base excision repair enzyme Fpg. Fpg cleaves DNA at oxidized bases and abasic sites, leading to SSBs. Closely spaced SSBs form DSBs that can be detected by PFGE. A striking increase in DSBs was found in PFGE using Fpg (Lane 4) as compared to PFGE without enzyme (Lane 2) (Figures 4A–4B). These results were also confirmed by using the human TK6 cell line (Figure 4B). Till now only high energy irradiation has been shown to generate OCDL which can subsequently be converted to the DSBs [31]. However, to the best of our knowledge, this is the first report showing the induction of OCDLs even at biologically relevant revels of H_2_O_2_ in human cells. In those previous studies co-localization of different base damage markers have been used to indicate clustered damage. During BER, the excision repair enzymes act to form a SSB as repair intermediate, therefore, the SSB markers e.g. XRCC1 would be preceded by enzymes or base damage markers e.g. OGG1, and not necessarily co-localized with them. Unlike high energy irradiation which cause a plethora of base damages in high quantities, low levels of oxidative stress would cause damage but in much less quantities. In addition, co-localization experiments are not able to reveal that the markers are within 20 base pairs (as per the definition for clustered DNA damage). Even 100–1000 bp difference would appear as same point during fluorescence experiments due to resolution issues.

H_2_O_2_ is known to exert its toxic effects through a Fe^2+^-mediated Fenton-type reaction [32]. The formation of more than one hydroxyl radical by closely spaced Fe^2+^ ions or through a redox cycling process may damage the DNA in close proximity, generating OCDLs. It is also possible that H_2_O_2_-induced SSBs in the DNA backbone may cause a localized denaturation in the vicinity of the break. This can further increase the probability of free radical mediated attack near that site as a result of loss of protective base stacking interactions. Another possibility can be when exogenous H_2_O_2_ inflicts an oxidative lesion near an already existing endogenous lesion on the opposite DNA strand, leading to DSBs during repair process.

### The NHEJ pathway plays a predominant role in ROS-induced mutagenesis

DSBs are one of the most toxic and mutagenic DNA lesions induced by ROS [33]. DNA DSBs are repaired *via* two general mechanisms: NHEJ and HR. While the HR pathway is usually error-free due to its dependence on homology, the NHEJ pathway is sometimes imprecise as it directly rejoins two severed DNA ends with deletions, insertions and frameshifts [34]. During NHEJ, the Ku heterodimer (Ku70/Ku80) binds to DSB ends in a sequence-independent manner and attracts DNA-PKcs to form the DNA-PK complex. DNA-PK then attracts the LIG IV complex (comprised of LIG IV, XRCC4 and XLF), which together seal the DNA ends [35]. This DSB repair pathway can lead to sequence alterations at the breakpoint when the ends are not compatible. The NHEJ pathway is active throughout the cell cycle, especially during the G0, G1 and early S phase. Our results showing H_2_O_2_-induced OCDLs/ DSBs and their repair by NHEJ, and the fact that NHEJ can sometimes be imprecise, directed us to investigate the role of NHEJ in oxidative stress-induced mutagenesis.

**Figure 5:**
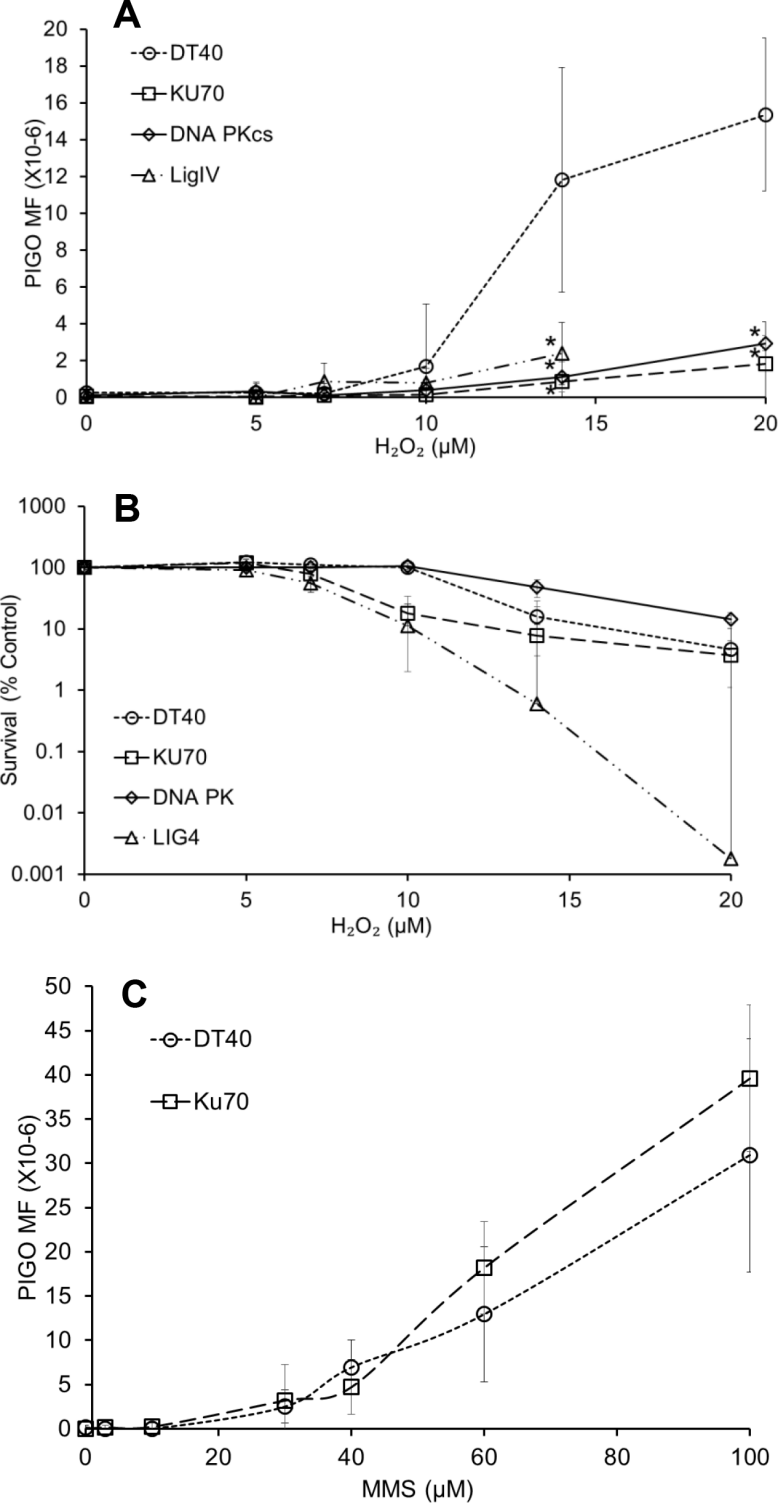
NHEJ plays a predominant role in oxidative stress-induced mutagenesis (**A**) PIG-O mutation analysis in wild type DT40 cells and NHEJ-deficient cells (*KU70-, LIGIV-, and DNA PKcs*-deficient) exposed to H_2_O_2_. *p < 0.05 when mutation levels were compared to wild type DT40 cells at respective doses using Student's *t*-test. (**B**) Survival of wild type DT40 cells and *KU70-, LIGIV-, and DNA PKcs*-deficient cells after exposure to H_2_O_2_, as assessed during the phenotype expression period in the mutation assay. (**C**) PIG-O mutation analysis in wild type DT40 cells and *KU70*- deficient cells exposed to methylmethanesulfonate. Data represent mean (*n* = 3) ± SD.

We employed a PIG-O mutation assay developed by our group [36]8 in the DT40 cell line and in NHEJ-deficient cells (*KU70-, LIGIV-*, and *DNAPKcs*-deficient cells). This assay was used because it can provide important information regarding the mechanisms of mutagenesis caused by H_2_O_2_ when performed in a series of mutant DT40 cells. Cells were exposed to 5–20 μM of H_2_O_2_ for 30 min, which is compatible with conditions of 8-oxodG and OCDL analysis. Compared to wild type cells, there was a drastic resistance in the increase in PIG-O gene mutations in all NHEJ deficient cells upon exposure to H_2_O_2_ (Figure 5A). It is worth mentioning that the levels of 8-oxodG in the *Ku70*-deficient cells were equivalent to that seen in DT40 cells, suggesting that similar levels of oxidative stress exist in both cell lines after H_2_O_2_ exposure (Figure 5A). To rule out any possibility of high cytotoxicity contributing to decreased mutation frequency, we addressed cell toxicity caused by H_2_O_2_ during the phenotype expression period (period between exposure to the H_2_O_2_ and selection agent) in the mutation assay. While *LigIV* and *Ku70*-deficient cells showed severe sensitivity to H_2_O_2_, *DNAPKcs*-deficient cells were less sensitive than wild type cells (Figure 5B). Irrespective of the differential H_2_O_2_ sensitivities, the effect on mutation frequencies in the absence of any of these NHEJ proteins was consistent and demonstrated a strong decrease in mutational events. To examine whether this decrease in mutations owing to NHEJ deficiency is also observed in another type of DNA damage that is repaired by the BER pathway (as in the case of H_2_O_2_), we examined the effect of NHEJ deficiency on MMS-induced PIG-O mutations (Figure 5C). No significant difference was observed between the mutation frequency of DT40 cells and *Ku70*-deficient cells exposed to MMS, demonstrating that the error-prone NHEJ pathway does not influence the mutagenesis caused by methylated DNA damage. The discrepancy in the methylated and oxidative stress-induced mutagenesis regarding NHEJ involvement might be attributed to the different modes of DNA lesions induced by MMS and H_2_O_2_, respectively. In the case of oxidative DNA damage, there can be a variety of complex DNA ends including oxidized deoxyriboses and 3′-deoxyriboses that first need to be processed for the repair to be completed. The methylated DNA lesion is not complicated to process, and formation of OCDLs may not occur, unlike ROS-induced damage. Due the possibility of the presence of DSBs with complicated ends, there is a possibility of NHEJ becoming activated in the case of oxidative, but not alkylated, damage. Hence, this may be the reason for differences observed in MMS and H_2_O_2_-induced mutation frequencies in NHEJ-deficient cells. Another important point worth mentioning is that it is generally assumed that, when one or more proteins in the classical-NHEJ pathway (*e.g.* Ku70, DNAPKcs, LigIV) are non-functional, the DSB repair occurs through a more error-prone alternative NHEJ pathway (*e.g.*, PARP1, XRCC3 and Lig3) [37]. However, it is only after eliminating the proteins of classical NHEJ (Ku70, DNA PKcs, LigIV), that we found a strong decrease in mutation frequency. This is also supported by our DNA repair data where KU70-mediated NHEJ and HR are acting as back-up for each other and in the absence of both the DSBs remain persistent.

### Biological relevance of ROS-induced OCDLs leading to NHEJ-mediated mutagenesis

Our results demonstrate that the error-prone NHEJ repair pathway, while trying to repair the DSBs caused via OCDLs, plays a pre-dominant role in oxidative stress-induced mutagenesis. Oxidative stress is linked to a long diverse list of human diseases- neurodegeneration, pulmonary diseases, carcinogenesis and other chronic inflammation-related disorders. Oxidative stress has also been suggested as the possible cause behind the inevitable process of aging.

We have utilized very low concentrations of H_2_O_2_ that are found in cells during acute and chronic inflammatory processes [22–24]. Moreover, under normal culture conditions, H_2_O_2_ is rapidly degraded within 30 min (exposure time in present study). Whereas during inflammatory conditions, there can be a continuous H_2_O_2_ flux from inflammatory cells making net exposure dose even higher. Based on our present data, we hypothesize that H_2_O_2_ causes DSBs *via* OCDLs formation and therefore may be an important regulator in inflammation-driven carcinogenesis. Redon et al., [38] found that the presence of a tumor may induce a chronic inflammatory response *in vivo* and observed that OCDLs were elevated in tissues distant from the tumor site due to inflammatory signals. A previous study reported an increase in frameshift mutations in human colorectal cells and demonstrated that oxidative stress as a result of chronic inflammation in the gastro-intestinal tract acted as the potential mutagen [39]. It is already known that mutations produced by NHEJ are of deletion/insertion and frameshift types. Taken together, these findings show that oxidative stress during inflammation is capable of causing OCDLs and DSBs. These DSBs, if unrepaired or misrepaired, can be relevant factors for cancer related to inflammatory processes.

Our results demonstrating the involvement of NHEJ in ROS-induced mutagenesis also share some similarities with a few previous studies in yeast cells. Heidenreich and Eisler [40] showed that the frequency of replication-independent frameshift reversions is reduced to ~50% in NHEJ-deficient yeast strains in the absence of any external exposure. Further investigations by Steinboeck et al. [41] revealed the presence of ROS and DSBs in these cells. The present data, combined with these observations in yeast, also suggest that NHEJ may be a source of spontaneous mutations due to endogenous DNA damage and oxidative stress. The imprecise nature of NHEJ that might cause spontaneous mutagenesis and its role in ROS induced DSBs also makes it relevant for aging. The “ROS-mutation” theory suggested by Lieber et al. [3] combines the effects of ROS on DNA damage and the slow loss of information by NHEJ (somatic mutations) as important factors for aging. Our results showing ROS-induced mutagenesis via NHEJ seem to fit this “ROS-Mutation” model of aging. Recently, mTOR pathway has also been proposed to play a role in aging. Our results showing ROS and mutagenesis can also be linked to the mTOR pathway through multiple links. For example, hydrogen peroxide activates the PI3-K/TOR/S6k pathway in human cells [42]. Different growth factors and UV induced peroxide has been shown to activate TOR [43, 44]. Activation of the PI3K/TOR pathway increases production of ROS, whereas inhibition of TOR decreases ROS levels [45, 46]. ROS can function as messengers in nutrient-sensing.as the amino acid leucine produces ROS to activate the TOR pathway [47].

ROS has been proposed to contribute to aging by damaging proteins and other biomolecules. Various signals including ROS activate the TOR pathway. TOR pathway has been linked inversely with life span extension in diverse organisms [48, 49]. TOR pathway inhibits autophagy and cause cell hypertrophy. Autophagy inhibits aging by degrading damaged proteins and organelles. Thus it might be a possibility that ROS or TOR independently or together act in the aging phenomenon [50]. From another perspective, ROS and mTOR may also have roles in tumorigenesis. Cancer cells with continuous activation of PI3K-mTORC1 signaling have autophagy inhibition which might lead to accumulation of damaged mitochondria and reactive oxygen species, which in turn can promote DNA damage and tumorigenesis [51].

In addition, p66Shc increases ROS production and shortens lifespan [52]. In drosophila, the neurofibromatosis-1 (NF1) gene mutants had elevated ROS production along with shortened lifespans [53]. However, p66shc and NF1 have also found to activate and deactivate the TOR pathway, respectively [54]. Therefore, p66shc and NF1 may be a common link/ factor in TOR and ROS pathway theories of aging.

The data presented here may also have significance for understanding the genomic instabilities in the non-proliferating cells of the body. Neurons have high levels of transcription and oxidative stress and remain in the G0/G1 phase. Consequently, neurons and other non-proliferating cells in human body will be mainly dependent on NHEJ for repair of DSBs, which in turn may contribute considerably to spontaneous mutagenesis. This idea is supported by the results from a previous study [55], which showed that migrating cortical neurons undergo oxidative DNA damage that is normally repaired by NHEJ. Failure to repair the DNA damage triggers neuronal apoptosis.

**Figure 6:**
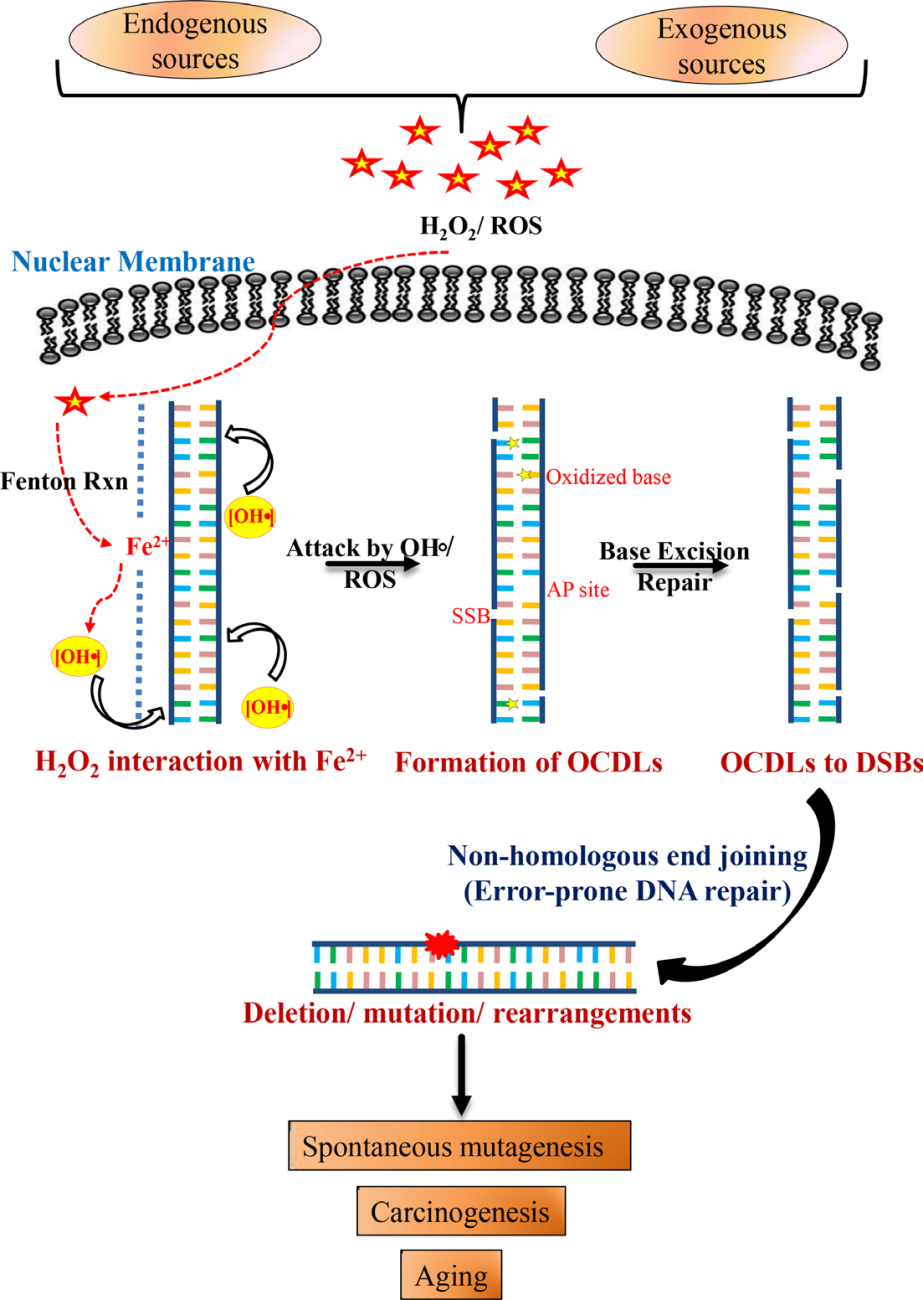
Schematic model for combined roles of OCDLs and error-prone NHEJ in oxidative stress-induced mutagenesis Oxidative stress and hydroxyl radicals can be produced in cells due to a variety of endogenous and exogenous factors. H_2_O_2_ even at low concentrations can react with Fe^2+^ that is weakly associated with the N7 of guanine (RTGR sequence; Fenton's reaction), leading to base damages and sugar lesions. The formation of more than one hydroxyl radical by closely spaced Fe^2+^ ions or the redox recycling process or during replication due to loose DNA structure may damage the DNA in proximity to form OCDLs. OCDLs can be converted to DSBs with complex DNA ends during the repair process. Since OCDLs can cause complex DNA ends, they may be repaired primarily by NHEJ (Ku70-, LIGIV-, and DNA-PKcs). However, the error-prone NHEJ repair pathway, while trying to repair these DSBs, may introduce mutations in the genome. Thus, the genomic instability produced may play relevant roles in the etiology of a wide variety of human diseases including cancer, chronic inflammation-related disorders, neuro-degeneration and aging.

In conclusion, the present study shows a predominant contribution of the NHEJ pathway in oxidative stress-induced mutagenesis. Our results indicate that oxidative stress even at low levels can cause clustered DNA damage that further leads to DSBs with complex DNA ends. Repairing such complex DSBs with NHEJ may result in mutations. This genomic instability can be involved in the etiology of a wide variety of human diseases including cancer. Our results are not only relevant for non-proliferating cells like neurons that have high ROS levels, but also suggest that ROS-induced and NHEJ-mediated mutagenesis may be critical for the production of spontaneous mutations and inflammation-related cancers (Figure 6).

## MATERIALS AND METHODS

### Materials

Penicillin/streptomycin, H_2_O_2_, methyl methanesulfonate (MMS), XTT, low melting agarose (LMA) and ethidium bromide were obtained from Sigma (St. Louis, MO). RPMI 1640 culture medium, Fetal bovine serum, phosphate buffer solution (PBS) and chicken serum were acquired from Invitrogen (Carlsbad, CA). Fpg was purchased from New England Biolabs (Beverly, MA). Fetal bovine serum (FBS) was obtained from Atlanta Biologicals (Norcross, GA). PA was purchased from University of Saskatchewan (Saskatoon, Canada). All DT40 mutants were derived from isogenic DT40 parent cell lines and cultured as previously reported [56]. All these mutant cells were originated in the same laboratory by Takeda group (Department of Radiation Genetics Graduate School of Medicine, Kyoto, Japan).

### DT40 DNA damage response analysis

DNA damage was determined by a 24-well plate-based DNA damage response analysis using a DT40 cell line and its mutants (Supplementary Table S1) knocked out in specific DNA repair as described previously [56]. Briefly, suspended cells (approx. 600 cells per 250 μl per well) were seeded into 24-well plates, exposed to H_2_O_2_ (0.5–3 μM) and allowed to divide for approx. 10 cycles. The H_2_O_2_ used was 30% aqueous and was serially diluted in sterile 1X PBS to obtain the appropriate concentrations in the plates. All H_2_O_2_ dilutions were made fresh and kept on ice. After cultivation, cell viability was determined by the XTT assay. The 95% confidence intervals is related to repeats within a single experiment with triplicate.

### Measurement of 8-oxodG by 2-dimensional liquid chromatography and mass spectrometry

Cells were exposed to H_2_O_2_ for 30 min, and DNA was extracted. DNA (20 μg) was enzymatically hydrolyzed to obtain a solution of free nucleosides. Quantitation of 8-oxodG was performed using a two-dimensional high performance liquid chromatography system coupled to a triple quadrupole mass spectrometer. An Agilent Technologies, Inc. (Santa Clara, CA) 1200 HPLC was used for sample injection and elution of 8-oxodG in the first dimension, and a Waters Corp. (Milford, MA) Acquity UPLC system was used for elution of 8-oxodG in the second dimension and for monitoring nucleosides by UV detection. Detection of 8-oxodG was performed with a Thermo Scientific (West Palm Beach, FL) Quantum Ultra triple quadrupole mass spectrometer (See Supplemental Information for details).

### PFGE for OCDL detection

To find an H_2_O_2_ exposure concentration in TK6 cells which is equivalent to exposure in DT40 cells (20 μM), the 8-oxodG and PIGO mutation data between TK6 cells [57] and DT40 cells were compared. Based on that, DT40 and TK6 cells (0.5 million/ml) were exposed to H_2_O_2_ at 20 and 40 μM respectively, for 30 min at 37°C followed by catalase (1.5 U/ml) addition to inactivate H_2_O_2_. For repair experiments in DT40 cells, a slightly higher concentration of H_2_O_2_ (50 μM) was used to accurately compare the DSBs signal (generation and repair) intensity. Sometimes the H_2_O_2_ exposure is given at 4°C to inhibit any potential repair and bring out the maximum damage. However, in our experiments we preferred 37°C as it represent the natural physiological state of cell in human body and shows the net damage (direct damage and repair enzyme-induced damage) despite efficient repair in the cellular system.

For PFGE, cells were embedded into LMA plugs (final cell number /plug was 0.5 million) as described previously [58]. Lysis was done at a low temperature (4°C) for 48 h to avoid any heat-induced artifactual damage as previously reported [59]. Further, to minimize oxidation artifacts during PFGE processing, all buffers were supplemented with 20 mM (2, 2, 6, 6- tetramethylpiperidin-1-yl)oxidanyl (TEMPO), a free radical spin trap and scavenger [60]. Plugs were washed four times in TE buffer (10 mM TRIS; 50 mM EDTA) and loaded onto a 0.8% agarose gel and separated by a CHEF-DR III pulsed-field electrophoresis system (Bio- Rad, Hercules, CA) for 24 h at 120° angle, 60–240 s, switch time, 4 Vcm^−1^. Gels were stained with ethidium bromide (0.5 μg/ml in double distilled water) for 30 min, destained, and an electronic image was obtained using a Fisher Scientific FBTI-88 transilluminator. Bands representing DSBs were quantified using Quantiscan (Biosoft, Cambridge, UK). Relative DSBs levels were obtained by comparing DSBs signals in each sample to the background signals observed for unexposed wild type DT40 cells.

For the detection of OCDLs an adaptation of PFGE [58] with further modifications was used with *Escherichia coli* repair enzymes, FPG, as damage probe. Briefly, after lysis and subsequent washing, the plugs were incubated with the repair enzyme for 2.5 h at 37°C followed by their inactivation with Proteinase K. The plugs were then washed with TE buffer before proceeding with electrophoresis.

### Immunofluorescence

TK6 cells (0.5 million/ml) were exposed to H_2_O_2_ (40 μM) for 30 min followed by addition of catalase. Cells were than fixed using 2% formaldehyde / PBS for 20 min, washed in PBS and were spotted on to double frosted microscopic glass slides (Fisher Scientific) at a concentration of 2 million/ml. Blocking was achieved using 5% (w/v) bovine serum albumin (BSA) in PBS-TT for 30 min. Cells were then incubated with 1:500 rabbit polyclonal anti-53BP1 antibody (Bethyl Laboratories, Montgomery, TX) and mouse monoclonal anti-cyclin A antibody (Abcam, Cambridge, MA) in 1% BSA/PBS-TT for 1–2 h at room temperature. Cells were then washed in PBS, incubated in 1:250 anti-mouse Alexa Fluor 647 (Life Technologies) and Alexa Fluor 488 antibodies (Jackson ImmunoResearch Laboratories, West Grove, PA) for 1 h. After washing with PBS, slides were mounted with a cover slip using Vectashield with DAPI (Vector Laboratories) and sealed using nail polish. Slides were analysed using confocal microscopy (Zeiss CLSM 700). Optical sections through the nuclei were captured at 0.5 μm intervals, and the images were obtained by maximum projection of the individual sections.

For BrdU experiments, cells were pulse-labelled with BrdU for 15 min immediately before exposure to H_2_O_2_ followed by co-exposure with H_2_O_2_. The rest of the procedure was followed as detailed above except the inclusion of the acid denaturation step to expose antigen. The antibodies used were anti-BrdU (Pierce antibodies) as primary and Alexa Fluor 555 (Life Technologies) as the secondary antibody. Each experiment was repeated thrice and approximately 50 nuclei/ experiment were counted.

### PIG-O mutation assay

The PIG-O mutation assay was performed as detailed in [36]. Briefly, wild type DT40 cells and NHEJ-deficient cells (Ku70; LigIV; DNAPKcs) were cultured for the mutation assay after re-populating the cells from small numbers to minimize background mutations. 1.25 × 10^6^/ml cells were exposed to different concentrations of H_2_O_2_ for 30 min at 37°C followed by catalase (1.5 U/ml) addition to inactivate H_2_O_2_. Cells (1. 25 × 10^6^ /10 ml) were seeded into 10-cm Petri dishes and allowed to cultivate for 24 h. After 24 h, the number of viable cells was quantitated by the Trypan Blue exclusion assay. Cells were maintained at 1.25 × 10^6^ cells/10 ml for 5 days to allow for phenotypic expression. After the phenotypic expression period was over, approximately 20,000 cells/50 ul/well were seeded in a 96-well plate in a medium containing 1.2 nM proaerolysin (PA). Cells from each dose were also plated into 96-well dishes at 1 cell/50 μL/well in the absence of PA to determine plating efficiency. All plates were incubated for approximately 7 days at 37°C, in 5% CO_2_, and in a humidified atmosphere. Subsequent colony formation was scored using an inverted microscope to determine the mutation frequency of each dose, calculated as described by Furth et al., [61].

### Statistical analysis

Statistical analyses were conducted with SPSS^®^ (v16.0, SPSS Inc.). Student's *t*-test and one-way analysis of variance (ANOVA) with Dunnet's test were employed to test for statistically significant differences between two groups and among multiple groups, respectively. For DDR analysis, survival data was log-transformed giving approximate normality. Analysis of covariance (ANCOVA) was used to test for mean intercept differences and differences in the slopes of the linear dose response curves between wild type and a series of mutant cells.

## SUPPLEMENTARY MATERIALS FIGURE AND TABLE


