# Supplementary material for: Oxidative stress at low levels can induce clustered DNA lesions leading to NHEJ mediated mutations
Source: Oncotarget. 2016 Mar 23;7(18):25377–90. doi: 10.18632/oncotarget.8298 (PMC5041911; doi:10.18632/oncotarget.8298)
Supplement: Supplementary file 1 [file oncotarget-07-25377-s001.pdf]

Oxidative stress at low levels can induce clustered DNA lesions leading to NHEJ mediated mutations

Supplementary Materials

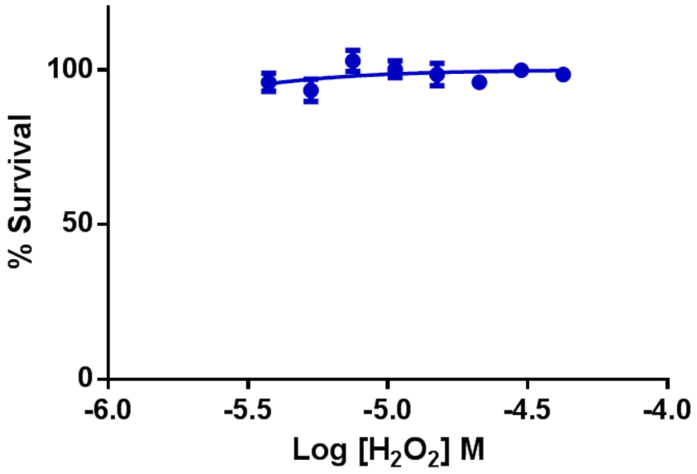

Supplementary Figure S1: Cell survival data for exposure conditions used in 8-oxodG analysis.

Supplementary Table S1: DT40 mutant cells used in this study

Gene	Function	References
RAD54	Homologous recombination (HR)	[1]
RAD51c	Homologous recombination	[2]
XRCC2	HR, promotion of Rad51 assembly	[2]
XRCC3	Homologous recombination	[2]
BRAC1	HR, damage checkpoint, transcription-coupled Base excision repair, regulation of transcription	[3]
FANCD2	Damage response to interstrand cross-links	[4]
KU70	Non-homologous end joining	[5]
LIGIV	Non-homologous end joining	[6]
RAD18	Regulation of translesion DNA synthesis (TLS), ubiquitin E3 ligase	[7]
REV1	Translesion DNA Repair, deoxycytidyl transferase activity	[8]
POLD3	Translesion DNA Repair	Unpublished (by Takeda et al.)
POL K	Translesion DNA Repair,	[9]
POL Theta	Translesion DNA Repair, Base excision repair	[10]
POL B	Base excision repair	[11]
FEN1	Base excision repair, processing of 5′ flap during DNA replication	[12]
PARP1	Poly(ADP-ribosyl)ation, BER, repair of DNA SSB and DSB	[13]
XPA	Nucleotide excision repair	[9]
MSH3	Mismatch repair	[14]
RAD9	Cell-cycle checkpoint control	[15]
RAD17	Cell-cycle checkpoint control	[15]
RAD54/ KU70	Homologous recombination/ Non homologous end joining	[5]
ATM	Cell-cycle checkpoint control	[16]

SUPPLEMENTARY EXPERIMENTAL PROCEDURES

Measurement of 8-oxo-dG by 2-dimensional liquid chromatography and mass spectrometry

Materials

8-oxo-dG analyte standard was purchased from Berry & Associates, Inc. (Dexter, MI). The stable isotope-labeled internal standard, [$^{15}N_5$] 8-oxo-dG, was purchased from Cambridge Isotope Laboratories (Andover, MA). Fisher Optima formic acid, acetic acid, and methanol were purchased from Fisher Scientific (Pittsburgh, PA). Ambion Turbo™ DNase was purchased from Life Sciences (Grand Island, NY). Phosphodiesterase I (0.74 U/bottle), alkaline phosphatase (10,000 U/bottle), and deferoxamine mesylate were purchased from Sigma-Aldrich (St.Louis, MO). Deionized water was generated on site using a filtration system from Pure Water Solutions (Hillsborough, NC).

DNA Hydrolysis

Samples of isolated DNA were enzymatically hydrolyzed to obtain a solution of free nucleosides. About 20 µg DNA were placed into polypropylene autosampler vials and diluted in a solution containing 100 µM deferoxamine mesylate, 10 µl of 10× Turbo™ DNase buffer (proprietary mixture, pH 7.5), and 250 fmol [$^{15}N_5$] 8-oxo-dG internal standard. Hydrolysis was initiated by addition of 16 U Turbo™ DNase I and incubation at 37°C for 30 min. Hydrolysis was completed by addition of 2.1 mU phosphodiesterase I, 1.6 U alkaline phosphatase and incubation at 37°C for 60 min. The final volume of each sample containing all reagents and internal standard was 100 µl. Samples were then injected onto the 2D-LC/MS system.

Quantitation of 8-oxo-dG by 2D-LC/MS

Quantitation of 8-oxo-dG was performed using a two-dimensional high performance liquid chromatography system coupled to a triple quadrupole mass spectrometer. An Agilent Technologies, Inc. (Santa Clara, CA) 1200 HPLC was used for sample injection and elution of 8-oxo-dG in the first dimension, and a Waters Corp. (Milford, MA) Acquity UPLC system was used for elution of 8-oxo-dG in the second dimension and for monitoring nucleosides by UV detection. Detection of 8-oxo-dG was performed with a Thermo Scientific (West Palm Beach, FL) Quantum Ultra triple quadrupole mass spectrometer. The first dimension of HPLC used gradient elution on an Agilent Poroshell C18 3.0 mm × 50 mm column with 2.7 um particles. The mobile phase consisted of 0.1% formic acid in water and methanol. Methanol composition was changed linearly from 2% at 0 min to 8% at 10 min, increased to 80% over 1 min, held at 80% for 4 min, decreased to 2% over 1 min, then held at 2% for 4 min for a total run time of 20 min. Eluate flowed through the UV detector until 7.7 min when an automated valve switched flow in line with the second chromatography column. 8-oxo-dG eluted onto the second column until the automated valve switched back to its original position at 9.2 min. The flow rate in the first dimension was reduced from 0.4 mL/min to 0.2 mL/min between 7.6 min and 9.2 min then was increased back to 0.4 mL/min at 9.3 min. The flow rate was reduced while the two chromatography columns were in line to prevent exceeding system pressure limits. The second dimension of chromatography used isocratic elution on an Agilent Poroshell C18 3.0 mm × 50 mm column with 2.7 um particles. The mobile phase consisted of 0.05% acetic acid and 0.02% formic acid in water and methanol. Methanol composition was maintained at 20%, and the flow rate was 0.15 mL/min. 8-oxo-dG eluted from the second column at 11.5 min. A 95 µL injection volume was used for samples and standards.

Mass spectrometer parameters were optimized for sensitivity and set to the following values: nebulizer voltage of 3000 V in positive mode, nitrogen sheath gas pressure of 35 (arbitrary units), nitrogen auxiliary gas pressure of 30 (arbitrary units), ion sweep gas pressure of 1 (arbitrary units), capillary temperature of 285°C, collision cell argon pressure of 1.5 mTorr, and collision cell energy of 12 eV. The mass transition for 8-oxo-dG was 284 → 168 m/z, and the mass transition for the stable isotope-labeled internal standard, [$^{15}N_5$] 8-oxo-dG, was 289 → 173 m/z.

Calibration curves using solutions of 8-oxo-dG and [$^{15}N_5$] 8-oxo-dG internal standard in 100 µM deferoxamine in water were generated with each sample set. To construct calibration curves, the amount of [$^{15}N_5$] 8-oxo-dG was kept constant at 237.5 fmol per injection, while 8-oxo-dG amounts were 9.5, 23.75, 95, and 237.5 fmol. Linear regression curves were calculated using the peak area ratio of 8-oxo-dG to internal standard versus fmol 8-oxo-dG injected. The limit of quantitation for 8-oxo-dG was 9.5 fmol per injection. The 2′-deoxyguanosine (dG) amount was determined by comparison with dG calibration standards using UV detection at 264 nm.

REFERENCES

1. Bezzubova O, Silbergleit A, Yamaguchi-Iwai Y, Takeda S, Buerstedde JM. Reduced X-ray resistance and homologous recombination frequencies in a RAD54-/- mutant of the chicken DT40 cell line. Cell. 1997; 89:185–193.
2. Takata M, Sasaki MS, Tachiiri S, Fukushima T, Sonoda E, Schild D, Thompson LH, Takeda S. Chromosome instability and defective recombinational repair in knockout mutants of the five Rad51 paralogs. Mol Cell Biol. 2001; 21: 2858–2866.
3. Martin RW, Orelli BJ, Yamazoe M, Minn AJ, Takeda S, Bishop DK. RAD51 up-regulation bypasses BRCA1 function and is a common feature of BRCA1-deficient breast tumors. Cancer Res. 2007; 67:9658–9665.

4. Yamamoto K, Hirano S, Ishiai M, Morishima K, Kitao H, Namikoshi K, Kimura M, Matsushita N, Arakawa H, Buerstedde JM, Komatsu K, Thompson LH, Takata M. Fanconi anemia protein FANCD2 promotes immunoglobulin gene conversion and DNA repair through a mechanism related to homologous recombination. Mol Cell Biol. 2005; 25:34–43.
5. Takata M, Sasaki MS, Sonoda E, Morrison C, Hashimoto M, Utsumi H, Yamaguchi-Iwai Y, Shinohara A, Takeda S. Homologous recombination and non-homologous end-joining pathways of DNA double-strand break repair have overlapping roles in the maintenance of chromosomal integrity in vertebrate cells. EMBO J. 1998; 17:5497–5508.
6. Adachi N, Ishino T, Ishii Y, Takeda S, Koyama H. DNA ligase IV-deficient cells are more resistant to ionizing radiation in the absence of Ku70: Implications for DNA double-strand break repair. Proc Natl Acad Sci U S A. 2001; 98:12109–12113.
7. Yamashita YM, Okada T, Matsusaka T, Sonoda E, Zhao GY, Araki K, Tateishi S, Yamaizumi M, Takeda S. RAD18 and RAD54 cooperatively contribute to maintenance of genomic stability in vertebrate cells. EMBO J. 2002; 21:5558–5566.
8. Simpson LJ, Sale JE. Rev1 is essential for DNA damage tolerance and non-templated immunoglobulin gene mutation in a vertebrate cell line. EMBO J. 2003; 22: 1654–1664.
9. Okada T, Sonoda E, Yamashita YM, Koyoshi S, Tateishi S, Yamaizumi M, Takata M, Ogawa O, Takeda S. Involvement of vertebrate polkappa in Rad18-independent postreplication repair of UV damage. J Biol Chem. 2002; 277:48690–48695.
10. Yoshimura M, Kohzaki M, Nakamura J, Asagoshi K, Sonoda E, Hou E, Prasad R, Wilson SH, Tano K, Yasui A, Lan L, Seki M, Wood RD, et al. Vertebrate POLQ, POLbeta cooperate in base excision repair of oxidative DNA damage. Mol Cell. 2006; 24:115–125.
11. Tano K, Nakamura J, Asagoshi K, Arakawa H, Sonoda E, Braithwaite EK, Prasad R, Buerstedde JM, Takeda S, Watanabe M, Wilson SH. Interplay between DNA polymerases beta and lambda in repair of oxidation DNA damage in chicken DT40 cells. DNA Repair (Amst). 2007; 6:869–875.
12. Matsuzaki Y, Adachi N, Koyama H. Vertebrate cells lacking FEN-1 endonuclease are viable but hypersensitive to methylating agents and H2O2. Nucleic Acids Res. 2002; 30:3273–3277.
13. Hochegger H, Dejsuphong D, Fukushima T, Morrison C, Sonoda E, Schreiber V, Zhao GY, Saberi A, Masutani M, Adachi N, Koyama H, de Murcia G, Takeda S. Parp-1 protects homologous recombination from interference by Ku and Ligase IV in vertebrate cells. EMBO J. 2006; 25:1305–1314.
14. Nojima K, Hochegger H, Saberi A, Fukushima T, Kikuchi K, Yoshimura M, Orelli BJ, Bishop DK, Hirano S, Ohzeki M, Ishiai M, Yamamoto K, Takata M, et al. Multiple repair pathways mediate tolerance to chemotherapeutic cross-linking agents in vertebrate cells. Cancer Res. 2005; 65:11704–11711.
15. Kobayashi M, Hirano A, Kumano T, Xiang SL, Mihara K, Haseda Y, Matsui O, Shimizu H, Yamamoto K. Critical role for chicken Rad17 and Rad9 in the cellular response to DNA damage and stalled DNA replication. Genes Cells. 2004; 9:291–303.
16. Morrison C, Sonoda E, Takao N, Shinohara A, Yamamoto K, Takeda S. The controlling role of ATM in homologous recombinational repair of DNA damage. EMBO J. 2000; 19:463–471.
